# Additive Manufacturing of Si-Added 7075 Aluminum Alloys: Microstructural, Mechanical, and Electrochemical Properties via Heat Treatment

**DOI:** 10.3390/ma18071544

**Published:** 2025-03-28

**Authors:** Gahyun Choi, Hobyung Chae, You Sub Kim, Soon-Ku Hong, Eunjoo Shin, Soo Yeol Lee

**Affiliations:** 1 Department of Materials Science and Engineering, Chungnam National University, Daejeon 34134, Republic of Korea; chlrkgus120@kaeri.re.kr (G.C.); usube2012@o.cnu.ac.kr (Y.S.K.); soonku@cnu.ac.kr (S.-K.H.); 2 Neutron Science Division, Korea Atomic Energy Research Institute, Daejeon 34057, Republic of Korea; hob2021@kaeri.re.kr (H.C.); it-sej@kaeri.re.kr (E.S.)

**Keywords:** selective laser melting, AA7075, precipitation, mechanical properties, electrochemical properties

## Abstract

Al 7075 alloy (AA7075) exhibits excellent strength yet poses significant challenges for additive manufacturing (AM) due to its complex composition and propensity for defects during rapid solidification. To address these issues, this study introduces a novel AA7075 containing a small amount of Si fabricated by selective laser melting (SLM). Despite concerns about reduced melt-pool stability at low Si content, the alloy was successfully processed into defect-minimized samples. Systematic evaluations of as-built and heat-treated (direct aging, solid-solution, T6) samples revealed distinct microstructural evolution and clear improvements in mechanical properties and corrosion resistance. Specifically, as-built and direct aging conditions showed high strength but limited ductility and pronounced galvanic corrosion due to inhomogeneous microstructures. Conversely, solid-solution and T6 treatments effectively homogenized the microstructure, significantly enhancing ductility and reducing corrosion susceptibility, with the T6-treated samples exhibiting the most balanced mechanical and electrochemical performance. By maintaining a favorable microstructural balance while minimizing Si-induced brittleness, the low-Si AA7075 demonstrates improved SLM processability and robust performance. These findings offer a new pathway for optimizing AM aluminum alloys through tailored heat treatments.

## 1. Introduction

Al 7075 alloy (AA7075), which contains relatively high amounts of Zn, Mg, and Cu, has long been used in the aerospace, automotive, and defense industries due to its outstanding strength and lightweight properties [1,2]. Nevertheless, attempts to apply AA7075 in additive manufacturing (AM) processes have yielded limited results in numerous studies owing to problems such as microcracks, residual stress, and process instability [3,4,5]. In laser-based AM, particularly the selective laser melting (SLM) process, AA7075’s high reflectivity and complex solidification behavior originating from mixed alloying elements hinder its ability to form a stable melt pool [6,7,8]. Consequently, it has been repeatedly noted that an uneven microstructure remains after construction, making it extremely challenging to achieve both robust mechanical properties and corrosion resistance [9,10].

By contrast, Al-Si alloys (e.g., AlSi10Mg), which typically contain high Si content (in the order of 7–12 wt%), have been the most extensively investigated materials in metal additive manufacturing [11,12,13]. The previous literature has repeatedly shown that various laser parameter optimizations, heat treatments, and post-processing techniques can be applied to Al-Si alloys to obtain a fairly uniform microstructure and satisfactory mechanical strength and corrosion resistance [14,15,16,17]. However, a fundamental drawback is that Al-Si alloys generally have lower absolute strength than traditional high-strength AA7xxx-series alloys, and they often fail to meet certain requirements [18,19]. For this reason, many studies in the aerospace and defense fields have tried to explore the development of additively manufactured high-strength alloys like AA7075. However, the aforementioned issues such as microdefects and thermal instability have prevented the achievement of satisfactory results.

In more recent work, researchers have partially attempted to combine the strengths of AA7075 and Al-Si alloys by adding an appropriate amount of Si to AA7075 to enhance flowability while retaining high-strength characteristics. Some studies have added Si to improve processability but ended up producing alloys closely resembling Al-Si systems, thus failing to fully utilize AA7075’s intrinsic mechanical advantages [20,21]. Moreover, as the Si content increases, excessive silicide (Si-based phases) is formed during solidification, leading to an increase in microscopic pores or brittle phases within the material, which, in turn, can degrade its performance [22,23]. Furthermore, many of these investigations have focused primarily on microstructure observations and simple tensile tests without systematical assessments such as for corrosion resistance or electrochemical behavior under actual service conditions.

To overcome these limitations, the present study aimed to successfully fabricate SLM specimens using an AA7075 powder containing only about 3.5 wt% Si (significantly less Si than in typical Al-Si alloys of 7–12 wt%), thereby maintaining a lower Si level while still employing SLM. Several recent studies have attempted to use lower silicon content in aluminum alloys to maintain high strength while improving additive manufacturability. Wang et al. reported the feasibility of fabricating low-Si AA7075 alloys via SLM, highlighting the importance of optimized energy input for minimizing defects such as porosity and solidification cracking [24]. Similarly, Montero-Sistiaga et al. (2016) showed that slight modifications in alloy composition could significantly improve SLM processability without sacrificing mechanical performance [25]. These studies underscore the necessity of carefully controlling the processing parameters to achieve optimal microstructures. However, comprehensive investigations simultaneously covering systematic microstructural evolution, mechanical performance, and electrochemical corrosion resistance in significantly low-Si AA7075 alloys fabricated via SLM remain scarce. Thus, this study aims to bridge this gap by systematically evaluating these critical aspects under various heat treatment conditions. This approach is a challenging endeavor intended to narrow the gap between existing Al-Si additive research and AA7xxx-series studies while enabling the more precise fabrication of high-strength alloys. Notably, because the Si content is not high enough to induce significant eutectic formation, a part of the inherent strengthening effect of AA7075 can be preserved; yet this also raises the risk of insufficient flowability or solidification stability, which can lead to microdefect formation.

In addition, this work carried out a comprehensive property assessment, encompassing not only mechanical properties but also corrosion resistance via electrochemical polarization tests as well as measurements of specimen thickness and area changes to determine actual corrosion-induced losses. AA7075 has long been acknowledged to be susceptible to corrosion, and the inherently heterogeneous microstructures and porosity arising from additive manufacturing may accelerate galvanic or localized corrosion. Nonetheless, previous research has often dealt with corrosion assessments limited to Al-Si alloys or provided insufficient corrosion data in the few attempts made with additively manufactured AA7075 [26,27]. Hence, by linking the microstructures of low-Si AA7075 to their mechanical and electrochemical responses, this study offers a broader framework that can contribute to future advances in high-strength AM materials design.

This work uses the SLM process with a powder containing a significantly lower Si level (3.5 wt%) than in common Al-Si alloys to tackle the challenges of additively manufacturing AA7075. It comprehensively evaluates both mechanical characteristics and electrochemical corrosion behavior. Furthermore, it systematically examines how multiple post-build heat treatments (as-built, direct aging, solid-solution, T6) influence the microstructure, precipitate evolution, tensile performance, and corrosion resistance, providing crucial insights into expanding the additive manufacturing feasibility of AA7075.

## 2. Material and Methods

### 2.1. Material

In this study, specimens were fabricated by SLM using a gas-atomized AA7075 powder to which 3.5 wt% Si had been added. The powder primarily consisted of spherical particles with a D_50_ of about 33 μm, exhibiting a uniform mixture of the base metal (Al) and alloying elements (Zn, Mg, Cu, Si), and its chemical composition is presented in Table 1. The SLM equipment used was Metalsys250, and throughout the fabrication process, both the powder supply system and the build platform were maintained in an inert atmosphere (high-purity Ar) to minimize oxidation and contamination.

For the SLM process parameters, the laser power was varied from 270 W to 850 W, while the scan speed was set between 750 mm/s and 850 mm/s and the hatching density ranged from 100 mm^2^ to 140 mm^2^. Each layer was deposited at a thickness of 30 μm, and the laser scanning strategy employed was a stripe pattern. The build platform was preheated as necessary, and the as-built printed material was fabricated as a block with a dimension of 150 mm × 15 mm × 18 mm. From this block, all the specimens were machined using electro-discharge machining under identical conditions to minimize variability among the samples. Tensile test specimens were produced according to ASTM E8 standards, with the sample’s longitudinal axis (LD) parallel to one of the scanning directions (150 mm direction) and its thickness direction aligned parallel to the build direction (BD).

Once the specimens were machined from the block, they were classified into four conditions depending on the heat treatment (Table 2). The first condition was the as-built state (denoted as AB), in which no heat treatment was applied after SLM. The second condition was a direct aging (DA) treatment without solutionizing, where the specimens were held at 120 °C for 24 h and then air-cooled to induce precipitation. The third condition involved only a solid-solution treatment (SS), where the specimens were kept at 470 °C for 1 h and then water-quenched to preserve the supersaturated solid solution. Finally, the fourth condition was T6, in which the same solution treatment (470 °C, 1 h, water quenching) was followed by aging at 120 °C for 24 h (air cooling) to maximize precipitation hardening. All heat treatments were performed in a vacuum furnace at pressures below 10^−3^ torr.

### 2.2. Characterization

The chemical compositions of the AA7075 powder and the SLM-fabricated samples were analyzed using the inductively coupled plasma (ICP, ICP-OES5110 of Agilent, Santa Clara, CA, USA) technique. For the microstructural observations of each heat-treated specimen, analyses were carried out using optical microscopy (OM, BX51M of Olympus, Tokyo, Japan), scanning electron microscopy (SEM, Merlin compact of Carl Zeiss, Oberkochen, Germany), electron backscattered diffraction (EBSD, Aztec HKL_NordlysNano EBSD of Oxford, UK), X-ray diffraction (XRD, SmartLab of Rigaku, Tokyo, Japan), transmission electron microscopy (TEM), and scanning transmission electron microscopy (STEM, JEM-ARM200F of JEOL, Tokyo, Japan). Prior to microstructural characterization, the samples were mechanically polished with a 0.04 µm colloidal silica solution, followed by electro-polishing in a 5% perchloric acid solution at 40 V for 3 s. The overall macrostructure, interlayer defects, and pore distribution were initially examined via OM. Using EBSD (operating at 20 kV with a step size of 0.76 µm), inverse pole figure (IPF) maps and kernel average misorientation (KAM) maps, indicating local deformation, were obtained. XRD was then employed to cross-validate the microstructural features. For the precipitate observation, TEM samples were prepared using a focused ion beam (FIB), and TEM was used to identify the phases of key precipitates. Additionally, the local chemical composition and distribution of the precipitates were measured by energy dispersive spectroscopy (EDS) attached to a CS-corrected STEM.

Tensile tests were performed at room temperature on a universal testing machine (UTM, MINOS of MTDI, Daejeon, Republic of Korea) at a fixed strain rate of 3.8 × 10^−4^, using an extensometer with a gauge length of 10 mm. Tensile tests were conducted in triplicate, and the average values along with their standard deviations for yield strength (YS), ultimate tensile strength (TS), and elongation are reported. After testing, the fracture surfaces were observed using SEM to analyze the fracture mechanisms. Vickers hardness (HM-200 of Mitutoyo, Kawasaki, Japan) was measured by taking ten readings per sample at different points and reporting the average.

Corrosion resistance was evaluated by electrochemical methods as well as actual dimensional changes in the samples. The electrochemical tests were carried out in a 1 M NaCl solution using a potentiostat (three-electrode configuration: working electrode as the specimen, Ag/AgCl reference electrode, and Pt counter electrode). The working electrode had an exposed area defined as a circular region with a diameter of 20 mm. After monitoring the open-circuit potential (OCP), potentiodynamic polarization was conducted for 1 h with a scan rate of 1.666 mV/s over an applied potential range of −2 V to +2 V. Tafel analysis was then applied by extrapolating the anodic and cathodic polarization curves to quantitatively derive the corrosion potential (E_corr_) and corrosion current density (I_corr_). Additionally, upon completion of each test, thickness reduction was determined by analyzing cross-sectional images obtained via optical microscopy to measure the overall decrease in the sample thickness resulting from corrosion. In parallel, area loss was quantified using Image J software (Version 1.54j) to assess the reduction in the cross-sectional surface area due to corrosion. This approach offers a straightforward and effective means of comparing corrosion resistance. All heat treatment conditions were evaluated using identical methods, enabling a comparative analysis of corrosion behavior across the different treatments.

## 3. Results and Discussion

### 3.1. Microstructure

**Figure 1:**
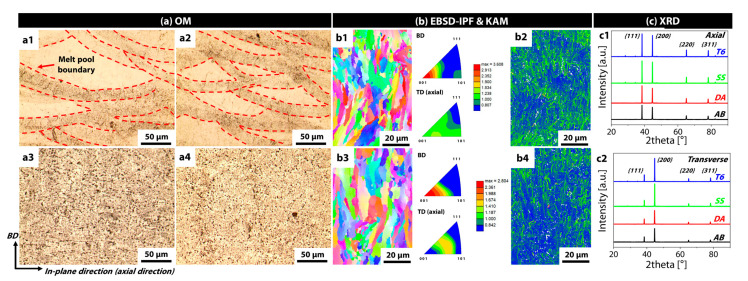
(**a**) Optical micrographs of each sample ((**a1**): AB, (**a2**): DA, (**a3**): SS, (**a4**): T6); (**b**) EBSD IPF maps and KAM maps ((**b1**,**b2**): AB; (**b3**,**b4**): SS); (**c**) XRD spectra measured in the (**c1**) axial (perpendicular to the build direction) and (**c2**) transverse (parallel to the build direction) orientations for each sample (black: AB, red: DA, green: SS, blue: T6).

The present study primarily focused on microstructural observations and precipitate analysis to characterize how the Si-added AA7075 manufactured by SLM changes under various heat treatment conditions. The results indicate that a solid-solution treatment performed during the heat treatment processes plays a pivotal role in determining the microstructure (Figure 1). Specifically, the AB and DA specimens exhibit similar characteristics, whereas the SS and T6 specimens share a distinct set of common features. As shown in Figure 1a, both the AB and DA specimens reveal pronounced melt-pool boundaries, formed during the additive manufacturing process, and relatively clear heat-affected zones (HAZ) are observed between layers (inset a1, a2). In contrast, such melt-pool boundaries are scarcely visible in specimens subjected to the solid-solution and T6 treatments, suggesting that the alloying elements have been rearranged and the microstructure has been homogenized under the high-temperature solid-solution conditions (inset a3, a4). Moreover, it appears that the aging treatment itself does not significantly alter the morphological features of the samples.

To quantitatively examine the grain size and deformation state of the microstructure, EBSD-IPF and KAM maps were examined (Figure 1b). In the as-built specimen, the average grain size was approximately 19 μm, and columnar grains grew with strong {100} texture along the build direction (inset b1). The geometrically necessary dislocation (GND) density estimated from the KAM map using OIM software (Version 8) reached about 3.8 × 10^13^ m^−2^, a relatively high value. Notably, the KAM map showed areas of elevated KAM values aligned with the melt-pool boundary (inset b2). In contrast, the SS specimen exhibited a slightly larger grain size of around 24 μm but did not show significant morphological changes compared to the as-built sample (inset b3). However, it revealed a similar preferred orientation distribution to the as-built sample. Its dislocation density decreased to 3.2 × 10^13^ m^−2^ (inset b4), indicating that some degree of recrystallization or dislocation annihilation occurred during the high-temperature solution treatment, thereby reducing residual deformation within the grains.

The XRD results (Figure 1c) revealed a distinct diffraction peak corresponding to the FCC aluminum matrix in all the specimens along with a few minor peaks attributable to precipitates. When measured in the axial direction (perpendicular to the build direction, inset c1), the intensities of the {111} and {200} peaks were relatively similar. However, in the transverse direction (parallel to the build direction, inset c2), the {200} peak was markedly stronger, indicating an overall {100} texture aligned parallel to the build direction. This observation is consistent with the EBSD-IPF results (Figure 1b) and the columnar grain structure and {100} preferred orientation commonly reported in 3D additive processes [28,29,30,31]. However, no distinct change in texture intensity was observed under various heat treatments.

**Figure 2:**
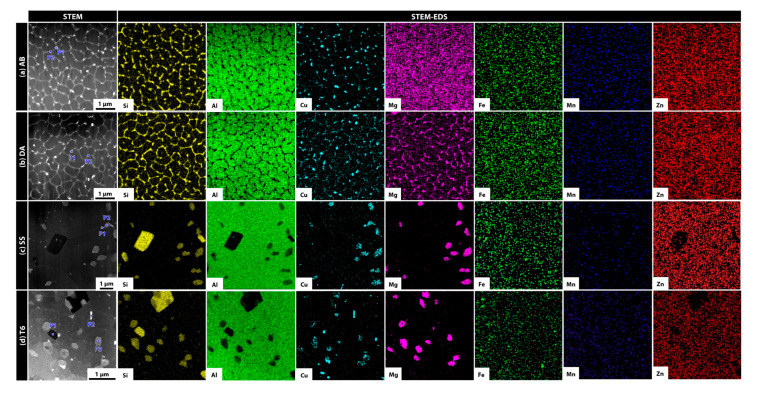
STEM-EDS micrographs illustrating the microstructure and elemental distribution maps for each sample.

The STEM and EDS analyses of the chemical composition showed prominent eutectic Si-rich network structures inside the melt-pool boundary of the AB and DA specimens (Figure 2a,b). In the DA sample in particular, Mg was also found to be enriched along these Si-containing networks, suggesting that Mg diffuses during aging through the fine solidification structure established in the as-built state. By contrast, in the SS and T6 specimens, the high-temperature solid-solution process dissolved Si more uniformly into the aluminum matrix, largely eliminating the eutectic structure seen in the AB and DA samples (Figure 2c,d). Instead, Si, Cu, and Mg clustered into coarser precipitates in certain regions, likely because solute elements were rearranged during cooling and aging, precipitating at specific temperature intervals to form relatively large particles. The EDS point scanning results (at.%) for precipitates under different heat treatment conditions are summarized in Table 3. In the AB condition, precipitates (P2) showed high Si concentrations (82.79%), while the net-like region (P1) was Al-rich (74.92%) with minor amounts of Cu and Mg. After direct aging (DA), the precipitate (P2) composition revealed a more balanced Si (36.06%) and Mg (10.41%) content. For the SS and T6 conditions, further redistribution of Si and Mg was observed. Especially notable was the high Si enrichment (86.98%) in the enlarged T6-treated precipitate (P1) and the increased Mg content (20.42%) in another precipitate (P3). These observations clearly demonstrate the significant influence of heat treatment processes on elemental redistribution and phase evolution.

**Figure 3:**
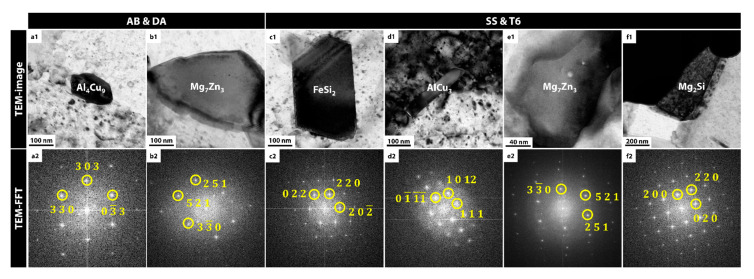
Morphology of the precipitates and their HRTEM-based FFT patterns observed in (**a1**–**b2**) AB and DA and (**c1**–**f2**) SS and T6.

In the AB and DA samples, the principal precipitates were found to be coexisting Al_4_Cu_9_ and Mg_7_Zn_3_ (Figure 3(a1–b2)). High-resolution TEM (HRTEM) observations and corresponding fast Fourier transform (FFT) patterns confirmed that Al_4_Cu_9_ has a cubic structure with a lattice parameter of about 8.703 Å. Mg_7_Zn_3_ also exhibited a cubic structure (a ≈ 14.17 Å), generally appearing as relatively larger dispersed particles compared to those containing Cu. There was no significant difference in the types of precipitates between these two samples. These high-strength phases beneficially contribute to tensile strength; however, when concentrated locally, they may trigger brittle fracture or heighten corrosion susceptibility.

The SS and T6 samples showed more complex changes in both the types and morphologies of the precipitates (Figure 3(c1–f2)). Among the residual phases that had not decomposed or dissolved at high temperature, FeSi_2_ was newly identified. It has an orthorhombic structure with lattice parameters a = 9.863 Å, b = 7.791 Å, and c = 7.833 Å. Additionally, AlCu_3_ (orthorhombic, a = 4.494, b = 5.189, c = 46.61), Mg_7_Zn_3_ (cubic, a = 14.17), and Mg_2_Si (cubic, a = 6.391) were observed in a more complexly distributed state. As Fe, Si, Cu, and Mg formed various intermetallic compounds during the solution and aging processes, the precipitate distribution became more diverse. Coarser particles are expected to influence ductility at fracture and indirectly affect corrosion mechanisms.

### 3.2. Mechanical Responses

The mechanical behavior of each heat-treated specimen (AB, DA, SS, T6) was primarily compared through hardness measurements and uniaxial tensile tests. Initially, Vickers hardness values showed 154 Hv and 168 Hv for the AB and DA specimens, respectively, whereas the SS and T6 specimens exhibited significantly lower values at 101 Hv and 105 Hv (Table 4). This decrease is presumably attributed to the high-temperature solid-solution treatment effect, which homogenizes the microstructure and increases grain sizes. Moreover, in the DA and T6 specimens, where aging was added to the AB and SS conditions, respectively, the hardness slightly increased, likely a result of nano-scale precipitate formation during the aging process.

**Figure 4:**
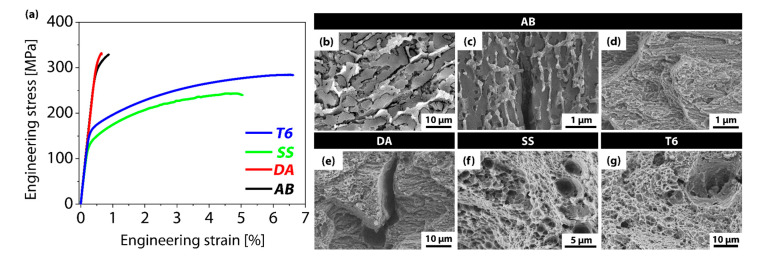
(**a**) Stress–strain curves of the AM-AA7075 samples subjected to various heat treatments (black: AB, red: DA, green: SS, blue: T6); (**b**–**d**) fracture surfaces of AB, (**e**) DA, (**f**) SS, and (**g**) T6.

A similar trend was distinctly observed in the tensile test results (Table 4, Figure 4a). The AB specimen showed a yield strength of 269 MPa, a tensile strength of 329 MPa, and elongation of 0.87%, while the DA specimen displayed 287 MPa, 332 MPa, and 0.65%, respectively. Both specimens shared a similar microstructure and were characterized by high strength but extremely low elongation. In contrast, the SS specimen, subjected to a solution treatment, exhibited a yield strength of 151 MPa and a tensile strength of 243 MPa, indicating a substantial reduction in strength, yet its elongation significantly increased to 5.0%. Subsequently, the T6 specimen, which underwent the same solution treatment as SS followed by the aging treatment, achieved a yield strength of 177 MPa, a tensile strength of 284 MPa, and elongation of 6.6%. This result demonstrates the ability to improve strength while maintaining high ductility relative to the SS specimen, which is a representative case of how aging can optimize the distribution and size of fine precipitates in the microstructure, providing reinforcement without severely compromising ductility.

Fractography observation showed that secondary cracks and quasi-cleavage fracture were prevalent in the AB and DA specimens (Figure 4b–e). In particular, microcracks tended to initiate and propagate around the net-shaped eutectic Si formed during the additive process, causing pronounced brittle behavior (Figure 4b,c). By contrast, the SS and T6 specimens, which underwent high-temperature solid-solution treatment to rearrange the microstructure, exhibited a dimple structure on the fracture surfaces, confirming that ductile fracture was the dominant mode (Figure 4f,g). This result is interpreted as arising from the more uniform dissolution of Si into the aluminum matrix during the solution treatment, along with finely dispersed precipitates formed during aging, which do not concentrate in localized regions, thus reducing potential crack initiation sites. Consequently, the SS specimen significantly improved elongation solely through the solution treatment, while the T6 specimen additionally achieved moderate precipitation strengthening, attaining a high ductility–strength balance.

### 3.3. Electrochemical Responses

**Figure 5:**
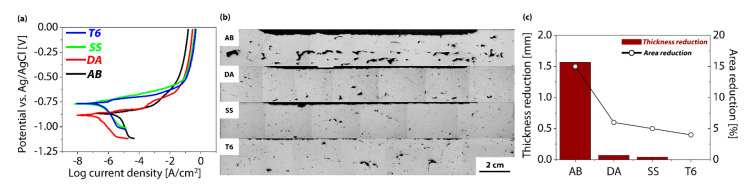
(**a**) Polarization curves of the AM-AA7075 samples under different heat treatments (black: AB, red: DA, green: SS, blue: T6), (**b**) morphologies of corroded cross-sectional surfaces, (**c**) thickness and area reductions for each sample.

The corrosion behavior of each heat-treated specimen (AB, DA, SS, T6) was quantified using electrochemical polarization experiments, yielding the E_corr_ and I_corr_. After testing, additional measurements of thickness and area loss were conducted to evaluate actual corrosion damage (Figure 5, Table 5). In the AB specimen, E_corr_ was measured at −0.855 V, while I_corr_ reached 3.345 × 10^−6^ A/cm^2^, a relatively high value. This finding is attributed to the inhomogeneous microstructures, pores, and locally concentrated compounds formed during the additive manufacturing process, all of which may trigger micro-galvanic corrosion and lead to an overall increase in corrosion activity [6,32]. As a result, thickness and area losses were recorded at 1.568 mm and 15%, respectively, representing the most severe corrosion observed among the four conditions.

The DA specimen exhibited a more negative corrosion potential of −0.883 V, yet its I_corr_ dramatically dropped to 0.189 × 10^−6^ A/cm^2^. Consequently, corrosion-induced thickness and area losses were 0.072 mm and 6%, respectively, showing a marked improvement compared to the AB condition. This result suggests that direct aging after additive manufacturing may partially redistribute the fine precipitates, thereby reducing or stabilizing potential sites for localized corrosion [33,34,35]. Although the measured potential is more negative, the reduced I_corr_ indicates that heat treatment-induced changes in the alloy’s microstructure have likely mitigated galvanic or pitting-type corrosion.

By contrast, the SS and T6 specimens, which underwent a high-temperature solid-solution treatment, exhibited corrosion potentials at −0.765 and −0.766 V, relatively more positive than those of the AB and DA samples, and correspondingly low corrosion current densities of 0.259 and 0.214 × 10^−6^ A/cm^2^. In particular, the T6 specimen displayed negligible thickness loss, and its area loss was only 4%, demonstrating outstanding corrosion resistance. This finding can be attributed to a more uniform redistribution of alloying elements within the matrix during the solution treatment, coupled with fine precipitates formed during aging that minimize pathways for localized corrosion. Although the SS specimen also exhibited an excellent corrosion resistance, reflected by a 0.039 mm thickness loss and 5% area reduction, it did not match the T6 specimen in terms of the degree of precipitate optimization, thus showing a slightly greater thickness loss.

Overall, it is evident that post-build heat treatments play a critical role in homogenizing the microstructure, reducing I_corr_, and minimizing dimensional changes due to corrosion. In particular, the T6 heat treatment effectively controls the size and distribution of fine precipitates, thereby improving both the mechanical properties and corrosion resistance; this outcome aligns well with previously reported mechanical responses, namely, enhanced ductility and sufficiently high strength.

### 3.4. Precipitation Behaviors

In this study, a variety of precipitates were observed to form in markedly different ways depending on whether the alloy was in the as-built (AB) state or had undergone subsequent heat treatments (direct aging, solid-solution, T6), and these variations proved decisive in determining both the mechanical behavior and electrochemical corrosion properties. First, in the AB and DA specimens, Al_4_Cu_9_ and Mg_7_Zn_3_ were predominantly observed (Figure 3). Al_4_Cu_9_, owing to its high Cu content, can enhance local strength but is distinctly hard and brittle, making it prone to serve as a crack-initiation site or to promote micro-galvanic corrosion [36]. Mg_7_Zn_3_ can similarly raise the risk of brittle fracture and increased corrosion susceptibility if it becomes unevenly coarsened [37]. In practice, both the AB and DA specimens exhibited extremely low elongation (less than 1%) relative to their high tensile strength (approximately 329–332 MPa), and electrochemical corrosion tests showed considerable I_corr_ along with the most severe thickness and area losses. This outcome represents a characteristic case in which overlapping eutectic Si-rich structures and high-strength precipitates induce localized galvanic reactions with a quasi-cleavage-dominated fracture mode.

On the other hand, FeSi_2_, AlCu_3_, and Mg_2_Si were formed as additional intermetallic phases in the specimens (SS, T6) that underwent a high-temperature solid-solution treatment, which uniformly redistributed the alloying elements. These compounds consist of FeSi_2_ (an orthorhombic phase arising from Fe and Si), AlCu_3_ (a Cu-rich phase), and Mg_2_Si (a well-known β-strengthening phase in Al-Mg-Si alloys), each varying in distribution and particle size according to the heat treatment conditions. While certain phases may become coarse and thus more prone to brittle fracture or corrosion, the dissolution of the net-like Si-rich eutectic structure during solution treatment, followed by finely dispersed precipitates generated through aging (T6), led to an overall improvement in ductility and corrosion resistance. Specifically, the SS specimen displayed an elongation increase to 5% and a substantial reduction in corrosion damage compared with AB and DA. Moreover, in the T6 specimen, the rearrangement of precipitates to more appropriate sizes further improved strength relative to SS, and electrochemical tests revealed minimal I_corr_ and dimensional changes, representing the best overall performance.

Localized EDS mapping after corrosion was not performed; therefore, a quantitative analysis of the selective corrosion behavior of the precipitates and the substrate could not be directly obtained. However, the overall corrosion performance, as evidenced by the measured thickness and area reductions, indicates that localized corrosion is predominantly driven by the galvanic coupling between the hard, brittle precipitates and the Al-rich matrix. These findings underscore the significant influence of heat treatment on microstructural redistribution and its consequential impact on corrosion behavior.

Hence, it becomes evident that the precipitates formed in AA7075 produced by additive manufacturing differ in both type and distribution via the heat treatment pathway, and this, in turn, results in balanced tensile properties and corrosion (galvanic, localized) behaviors. If, like the AB or DA specimens, the alloy contains numerous locally enriched high-strength precipitates, the material achieves high tensile strength but suffers from severe microcracking and corrosion. Conversely, homogenizing the microstructure through solution treatment and aging can block pathways for localized corrosion and maintain a suitable precipitate distribution, thereby restoring the strength to an acceptable level. Ultimately, this study demonstrates that controlling the formation and distribution of precipitates during heat treatment is crucial for balancing mechanical performance and corrosion resistance, which, in turn, provides essential guidelines for designing high-performance additive alloys and optimizing post-processing treatments.

## 4. Conclusions

This study systematically investigated how microstructure and precipitate changes induced by various heat treatments (direct aging, solid-solution, T6) influence the mechanical and corrosion properties of an AA7075 with low-added Si following additive manufacturing (as-built condition). In the as-built and direct aging samples, high-strength phases such as Al_4_Cu_9_ and Mg_7_Zn_3_ were locally enriched, achieving a high tensile strength of approximately 330 MPa. However, their inherently non-uniform microstructure including eutectic Si regions led to an extremely low elongation (below 1%) and frequent galvanic corrosion, suggesting potential issues of extremely limited ductility and severe corrosion resistance in actual component applications. In contrast, the specimens that underwent solution treatment and subsequent aging (T6) formed additional precipitates such as FeSi_2_, AlCu_3_, and Mg_2_Si yet still exhibited a uniform overall microstructure and evenly dispersed fine precipitates, thereby improving elongation (up to 6.6%), tensile strength (284 MPa), and corrosion resistance (minimal thickness and area losses). Notably, the T6 specimen demonstrated the best combined performance in both the mechanical and electrochemical tests, interpreted as the synergistic outcome of effectively eliminating the inhomogeneity from additive manufacturing via solution treatment and optimizing precipitate distribution through aging. Consequently, this work demonstrates that aging alone is insufficient to maximize the performance of low Si-added AA7075 additively manufactured components; rather, a suitable combination of solution treatment and aging is necessary to homogenize the microstructure and control the size and distribution of precipitates.

## Figures and Tables

**Table 1:** Nominal and measured chemical compositions of the wrought alloy, used powder, and as-built samples (wt%).

Sample	Si	Fe	Cu	Mn	Mg	Cr	Zn	Ti	Al
Wrought (nominal)	0.04	0.13	1.51	0.14	2.41	0.22	5.54	0.03	Bal.
Powder (measured)	3.45	0.13	1.46	0.13	2.32	-	4.54	-	Bal.
As-built (measured)	5.31	0.15	2.01	0.12	2.20	-	5.11	-	Bal.

**Table 2:** Summary of the sample conditions and corresponding heat treatment procedures (AB: as-built; DA: direct aging; SS: solid solution; T6: solution treatment followed by aging).

Abbreviation	Sample Condition	Description of Heat Treatment Process
AB	As-built	As-built condition (no additional heat treatment after SLM)
DA	Direct aging	Aging directly from as-built condition (120 °C, 24 h)
SS	Solid solution	Solutionized at 470 °C for 1 h, followed by water quenching
T6	T6 heat treatment	Solid-solution treatment (470 °C, 1 h), water quenching, and aging (120 °C, 24 h)

**Table 3:** EDS point scanning results in AB, DA, SS, and T6-treated AM-AA7075 samples (at.%).

Sample	Position	Si	Fe	Cu	Mg	Zn	Al	O
AB	P1	8.12	0.09	2.97	1.34	0.06	74.92	12.51
	P2	82.79	0.00	0.00	0.97	0.00	9.98	6.27
DA	P1	1.83	0.09	3.34	0.02	0.03	85.10	9.60
	P2	36.06	1.28	2.88	10.41	1.25	35.31	12.82
SS	P1	2.49	0.00	3.43	3.27	0.00	75.51	15.31
	P2	51.93	2.18	1.40	15.99	0.03	15.32	13.05
T6	P1	86.98	1.37	0.30	0.45	0.00	5.55	5.35
	P2	2.21	0.01	3.24	1.29	0.00	84.19	9.06
	P3	45.98	0.47	1.06	20.42	0.06	19.46	12.56

**Table 4:** Summary of the hardness and tensile test results for the AM-AA7075 samples under different heat treatment conditions.

Sample	Hardness [Hv]	Yield Strength [MPa]	Tensile Strength [MPa]	Total Elongation [%]
AB	154 ± 8.4	269 ± 11.1	329 ± 26.3	0.87 ± 0.45
DA	168 ± 5.9	287 ± 7.6	332 ± 20.9	0.65 ± 0.25
SS	101 ± 3.7	151 ± 9.2	243 ± 10.5	5.0 ± 1.24
T6	105 ± 4.5	177 ± 13.4	284 ± 14.2	6.6 ± 1.35

**Table 5:** Summary of the electrochemical test results for the AM-AA7075 samples with different heat treatment paths.

Sample	E_Corr_ [V]	I_Corr_ [10^−6^ A/cm^2^]	Thickness Reduction [mm]	Area Reduction [%]
AB	−0.855	3.345	1.568	15
DA	−0.883	0.189	0.072	6
SS	−0.765	0.259	0.039	5
T6	−0.766	0.214	0	4

## Data Availability

The original contributions presented in this study are included in the article. Further inquiries can be directed to the corresponding author.
